# Family-centered delirium prevention and treatment using video calls: the FACE Delirium trial

**DOI:** 10.1007/s41999-023-00854-2

**Published:** 2023-08-30

**Authors:** Johannes Trabert, Andreas Schenk, Rejane Golbach, Rupert Püllen, Sandra Schütze

**Affiliations:** 1 Department of Geriatric Medicine, AGAPLESION Markus Hospital, Wilhelm-Epstein-Strasse 4, 60431 Frankfurt, Germany; 2 Institute for Biostatistics and Mathematic Modelling, Faculty of Medicine, Goethe-University, Frankfurt, Germany

**Keywords:** Delirium, Prevention, Family, Video calls, Pandemic

## Abstract

**Purpose:**

In the FACE Delirium trial, we investigated the feasibility of a structured FAmily-CEntered delirium prevention and treatment during the corona pandemic.

**Methods:**

Patients hospitalized in a German geriatric medicine department were included in this single-center, prospective, single-arm feasibility study. Their relatives received a short training on delirium and volunteers or paid staff members facilitated video calls. The primary endpoint was reached when contact between patients and their relatives occurred on ≥ 80% of treatment days, either via video call or visit.

**Results:**

38 patients were included (age 83.0 ± 5.9 years; 73.7% women). 76.3% reached the primary endpoint. Due to the pandemic, 99.3% of the contacts were video calls with a duration of 24.8 ± 16.3 min.

**Conclusion:**

Family-centered delirium prevention and treatment using video calls is feasible among hospitalized geriatric patients. Daily implementation in clinical practice poses challenges and requires motivated and qualified staff.

## Introduction

Delirium is one of the leading complications affecting hospitalized geriatric patients, associated with higher mortality, longer hospital stays, reduced independence, permanent cognitive impairment, and higher healthcare costs [1–3]. In geriatric wards, delirium rates vary between 17 and 35% [1, 4], often with delirium already present on admission.

Non-pharmacological interventions are essential for prevention as well as treatment of delirium [5, 6]. Involving family members is particularly effective: in two independent studies, presence of family members trained in delirium prevention measures led to a delirium reduction of 59% and 86%, respectively [7, 8].

However, both studies required a continuous presence of relatives or caregivers for at least 6 h per day.

The studies were conducted in Chile and China, neither which are comparable to Western countries concerning population or family structures. Therefore, concepts applied in these two studies may not be transferable to Western nations. Furthermore, social restrictions and hospital visit limitations during the corona pandemic counteract this concept.

Alternatives to physical presence, such as video calls, provide a practical alternative. To date, there have been no prospective studies on implementation of family-centered delirium prevention or treatment using video calls.

The aim of the FACE Delirium trial is to investigate the feasibility of family-centered delirium prevention and treatment in hospitalized geriatric patients using video calls.

### Study design and methods

In this single-center, prospective, single-arm feasibility study, patients were recruited between March, 13th and May, 19th 2021 in the Department of Geriatric Medicine of the AGAPLESION Markus Hospital in Frankfurt, Germany. A total of 77 patients were screened.

Inclusion criteria were age ≥ 70 years and relatives willing to participate. Exclusion criteria were severe visual or hearing impairment as well as history of severe dementia, as these impairments were thought not to be compatible with conducting video calls. Patients with history of mild to moderate dementia or delirium on admission were not excluded.

After inclusion, relatives received a 20-min phone-based training on the subject of delirium, delirium-preventive, and therapeutic measures by a trained team of nursing staff (see Table 1).

**Table 1:** Measures of delirium prevention and treatment provided by family members

Encourage mobilization and activity (sitting at bedside, short walks)
Provide orientational clues to the patient (concerning place, time, situation)
Encourage wearing of glasses and hearing aids
Encourage fluid and food intake
Alert medical staff in case of signs of delirium

Every day, volunteers or paid staff members provided Samsung tablets at prearranged times, supported the initiation of video calls, and ensured good image and sound quality. Depending on relatives' preferences, Threema, Skype, or Whatsapp was used. Patients were encouraged to sit at bedside or on a chair during video calls.

Primary endpoint was successful participation in the study protocol. This was defined as contacts between patients and relatives on ≥ 80% of all treatment days, either via video call or visit. A video call had to last ≥ 5 min.

Since this was a feasibility study, we defined the following interpretations in advance: If overall participation of all relatives and patients is successful in

 < 40%: not feasible.

40–59%: feasible.

 ≥ 60%: well feasible.

Comprehensive geriatric assessment was obtained on all patients, including Barthel Index (BI), Mini-Mental Status Examination (MMSE), as well as tests of hearing (Whisper Voice test) and vision (Landolt ring chart). Additionally, we performed delirium screening on admission and discharge using 3D-CAM [9]. Diagnosis of delirium and dementia was obtained from the medical chart.

### Statistics

Patient data were documented using Microsoft Excel Version 2016 and statistics were analyzed with BiAS software (Version 11.12. After sample size calculation, 38 patients should be included. This number of cases is obtained using a binomial test with α = 5%, β = 80%, an assumed probability of successful participation by the relatives of 60% and a rejection of the feasibility of the concept for a proportion of 40%.

For testing the null hypothesis that the proportion of successful participants is less than 40%, a one-sided exact binomial test was used.

## Results

29 of 38 patients and their relatives (76.3%) met the primary endpoint of being in touch ≥ 80% of treatment days (p < 0.001). As a result, the null hypothesis that less than 40% of relatives and patients participate successfully could be rejected, and feasibility of family-centered delirium prevention and treatment could be inferred.

Since the study was performed during the second wave of the corona pandemic with an almost complete visiting ban in hospitals, almost all contacts were made via video calls. A total of 580 family interactions were documented on 667 cumulative treatment days, 576 (99.3%) being video calls, and only 4 (0.7%) being physical visits, indicating that family-centered delirium prevention and treatment is also well feasible using video calls.

Duration of video calls was 24.8 ± 16.3 min. The longest video call lasted 244.0 min. No side effects such as falls or agitation associated with video calls were observed.

One patient was isolated during the entire stay due to colonization/infection with a multi-resistant pathogen. Three patients were temporarily isolated as SARS-CoV-2-contact persons. Despite the isolation measures, these four patients successfully participated in the study.

2 ± 1.1 relatives per patient participated in the study. The farthest distance to a relative was 700 km. 7 (18.4%) patients had a history of mild to moderate dementia and 11 (28.4%) patients had delirium on admission.

Both MMSE and BI were not associated with study participation (Spearman's rank correlation). The same was shown for diagnoses of dementia or delirium in multivariable regression analysis.

9 of 38 patients (23.7%) did not successfully participate in the study with altogether 44 no-contact days. Most common reason for a no-contact day was non-reachable relatives in 28 of 44 cases (63.6%). In 12 out of 44 cases (27%), video calls lasted less than 5 min. Only 4 times out of 44 (9%), patients refused a video call.

Characteristics of all 38 included patients are listed in Table 2.

**Table 2:** Patient characteristics

Total number of included patients	38
Sex	
Female	28 (73.7%)
Male	10 (26.3%)
Age (mean $\pm$ SD)	83.0 $\pm$ 5.9 years
Number of relatives (mean $\pm$ SD)	2.0 $\pm$ 1.1
Distance to next relative (median, IQR)	5.0 [0.0, 20.0] km
Dementia diagnosis	7 (18.4%)
Delirium on admission	11 (28.9%)
Mini-mental state examination on admission* (mean $\pm$ SD)	24.7 $\pm$ 4.5
Mini-mental state examination at discharge* (mean $\pm$ SD)	25.4 $\pm$ 4.5
Barthel index on admission (mean $\pm$ SD)	42.9 $\pm$ 17.5
Barthel index at discharge (mean $\pm$ SD)	61.4 ± 19.0
Hearing impairment*	
None	30 (78.9%)
Mild	8 (21.1%)
Vision impairment*	
None	10 (26.3%)
Mild	28 (73.7%)
Isolated patients	4 (10.5%)
Duration of daily video calls (mean $\pm$ SD)	24.8 $\pm$ 16.3 min
Number of video calls/all contacts	578/582 (99.3%)
Number of in person visits/all contacts	4/582 (0.7%)
Total number of treatment days	667

*of 38 patients, the following values are missing (e.g., due to ad hoc discharge):

MMSE on admission: 4 values

MMSE at discharge: 2 values

Barthel Index at discharge: 2 values

Vision impairment: 1 value

## Discussion

The FACE Delirium trial demonstrates that family-centered delirium prevention is feasible in an acute geriatric ward in Germany, even if in person visits are not possible.

A broad spectrum of geriatric patients was represented in the study, including patients with history of mild to moderate dementia. Present delirium as well as relevant functional impairment and active isolation measures. Cognitive and functional impairment have been discussed to be important barriers for using video calls [10]. However, we were able to demonstrate otherwise in a challenging setting during the COVID-19 pandemic. Thus, we believe our results can be generalized to any geriatric setting.

Previous studies investigating family-centered delirium prevention showed feasibility as well as considerable reduction in delirium incidence. However, these studies presupposed physical presence of family members or caregivers [7, 8]. This is not impossible but much more difficult if isolation measures are in place [11]. Accordingly, the need for implementing alternative ways to keep in contact with relatives in hospital settings, e.g., by the use of video calls, was particularly highlighted during the corona pandemic. Patients were routinely confronted with medical staff and relatives dressed in personal protective equipment which may be a contributing factor to high delirium rates in elderly corona patients [12].

These important findings from the pandemic will be of ongoing interest because isolation measures continue to be necessary due to other infectious diseases or multi-resistant pathogens. A survey among family members of deceased veterans showed a clear need for the use of video calls in such a setting. Providing such alternatives to in person visits in times of visiting bans led to an improved perception of communication quality [13].

In a large multinational study among patients with COVID-19, family visits both in person and virtual were associated with a significantly lower risk of delirium. 96% of study sites reported encouraging virtual visits; however, they did not differentiate between video calls or regular phone calls [12].

The strength of the FACE Delirium trial is the combination of information on delirium and its potential consequences combined with reliable daily availability of video calls. We believe family members’ knowledge about delirium and its potential consequences implicated their strong motivation, which is reflected in the high participation rate. Awareness and knowledge of delirium was poor in a survey among family members of patients with delirium [14]. Since education on delirium symptoms has been shown to be effective [7, 8], it should be routinely provided to family members.

A potential bias for the high participation rate may be visiting bans which were in place in all hospitals in the region during the study. Isolation may have been a strong trigger for patients and their relatives to participate in the study.

A weakness of the study is the lack of data on the actual content of the individual video calls. It was not documented whether family members actually performed delirium-preventive measures while conducting the video call, such as helping with orientation. However, providing a visual contact with a loved one without the need of wearing a mask, combined with mobilization measures such as sitting upright while on the video call, might already pose as strong delirium-preventive measures.

Thomas et al. developed an easy to use, webex-based tool for video calls in an ICU setting in Australia. However, this tool also requires staff assistance and the feasibility or a clinical effect of the use of video calls has not been demonstrated thus far [15]. We provided different apps for video calls with Whatsapp actually being preferred by family members almost exclusively. Since Whatsapp is widely used, no special training was required for the staff.

Due to high workload in hospitals, it seems unrealistic for nursing staff to support all patients in a ward in conducting video calls. This was also our experience during the height of the corona pandemic with visiting bans in place. Thus, additional paid staff members and/or volunteers were required. However, in non-pandemic times, considerably fewer patients will be in need of video calls.

We believe it is especially worth focusing on patients with reduced activity, particularly those with hypoactive delirium or depression. In this population, we experienced a positive effect on mobilization: most patients were sitting during the video calls, even those who preferred to lie in bed outside of the stimulation by their relatives. However, since there was no clinical endpoint, our promising subjective experience could not be proven in our present study. Further studies with clinical endpoints are needed.

## Conclusion

The FACE Delirium trial showed that regular involvement of family members in delirium prevention and treatment is well feasible even when in person visits are not possible. 76.3% of patients and their relatives successfully participated in the study protocol, i.e., a video call or visit took place on ≥ 80% of all treatment days. Due to the corona pandemic, contacts were made almost exclusively via video calls. Although implementation of this measure is complex and personnel intensive, it makes overcoming barriers of isolation possible.

## Data Availability

Anonymised study data that support the findings of this study are available from the corresponding author, [JT], upon reasonable request, given a positive statement of an ethics board is present.
